# Factors Associated with Parents’ Adherence to Different Types of Exercises in Home Programs for Children with Disabilities

**DOI:** 10.3390/jcm8040456

**Published:** 2019-04-05

**Authors:** Carmen Lillo-Navarro, Joaquina Montilla-Herrador, Pilar Escolar-Reina, Silvana L. Oliveira-Sousa, Jose A. García-Vidal, Francesc Medina-Mirapeix

**Affiliations:** 1 Department of Pathology and Surgery, University Miguel Hernández, Sant Joan, 03550 Alicante, Spain; mclillo@goumh.umh.es (C.L.-N.); soliveira@umh.es (S.L.O.-S.); 2 Departament of Physiotherapy, University of Murcia, Campus de Espinardo, 30100 Murcia, Spain; pescolar@um.es (P.E.-R.); garciavidal@um.es (J.A.G.-V.); mirapeix@um.es (F.M.-M.); 3 Research group Fisioterapia y Discapacidad, Instituto Murciano de Investigación Biosanitaria-Virgen de la Arrixaca (IMIB-Arrixaca), El Palmar, 30120 Murcia, Spain

**Keywords:** exercise, parents, home programs, physiotherapy, adherence, early intervention

## Abstract

There is a lack of knowledge with regard to the adherence to different types of exercises prescribed for children with disabilities. The aim was to examine parents’ adherence to prescriptions of different types of home exercises; to identify associated factors related to the parents, the children and the environment, and to assess the relative influence of the behaviour of health professionals. Parents (393) were recruited from 18 early intervention centres. A cross-sectional survey using a self-reported questionnaire was used to examine whether three types of exercises (“flexibility exercises”, “neuromotor development training” and “body mechanics and postural stabilisation”) were prescribed in their home programs; if the child had received exercises according to a prescription; and items related to the parents, child, environment, and health professionals. The adherence rates were different among the types of exercises. Parents with low perception of barriers and high self-efficacy had a higher adherence to neuromotor development training and postural stabilization, whereas parents with a high level of knowledge increased their odds of adherence to flexibility exercises. Health professionals’ behaviour had a distinct influence on the adherence to different exercises. This study suggests the need to specifically consider the types of exercises prescribed in the management of adherence to home programs.

## 1. Introduction

The use of Home Exercise Programs (HEPs) for children with disabilities, is a widespread resource that often contributes to an increase in their amount of practice and functioning [1]. The HEPs prescribed for children are usually individualised, based on family goals, and generally include different types of exercises and interventions [2,3]. It is well known that adherence is a desirable and essential behaviour in order to achieve the goals of an HEP. However, parental adherence is estimated to be lower than 50% [4,5]. Nevertheless, as studies have typically measured parents’ adherence to HEPs as a whole and single construct [5], this percentage could vary for different exercises. According to evidence from qualitative research [6,7], it is suggested that instead of performing the whole home program, caregivers usually choose those activities that are easiest for them. For example, studies on cystic fibrosis have shown that there are different levels of adherence to different parts of a home therapeutic regime, such as pharmacological treatment, nutritional prescriptions and exercise [8]. 

Given the fact that there is a lack of knowledge regarding adherence according to different types of exercise [3], there is a need to study whether certain factors associated to the exercises prescribed in an HEP may influence adherence. The existing literature provides some insight into the different factors that influence adherence to different kind of exercises in adult populations [9]. Nevertheless, it has been suggested that adherence to children’s exercise regimes is influenced by factors that may not be relevant for adults [10], as more complex relations [11] and circumstances appear simultaneously among children, parents and health professionals. 

This study examined three issues in a population of children with or at risk of developmental disabilities attending paediatric services in early intervention centres: (1) to determine rates of parents’ adherence to different types of exercises featured in their child´s HEP; (2) to identify what factors related to the environment, the parents and the child receiving treatment are more likely to be associated to parents’ adherence to these types of exercises; (3) to assess the relative influence of the behaviour of health professionals on parents’ adherence to each type of exercise after making adjustments for associated environmental, parental and child-related factors.

## 2. Methods

### 2.1. Study Design and Participants

This study was a multicentre survey design using a self-administered questionnaire. The study obtained approval by the Ethics Committee of the University of Murcia (approval No. 129/05). All participants provided written informed consent prior to the data collection. The inclusion criteria consisted of parents of children aged between six months and six years with a prescribed HEP and attending early intervention centres in Murcia (Spain) over a period of six months at least. The exclusion criteria consisted of the inability to read or write in Spanish. 

### 2.2. Data Collection and Measurements 

The analysis developed in this paper is based on a self-report questionnaire (Table A1). The questionnaire included questions about components of HEP, adherence behaviours and potential associated factors. A previous qualitative study [7] was used to identify dimensions of healthcare professionals’ behaviours associated to parents’ adherence. On the basis of comments made by parents, several candidate questionnaire items were written for each domain. An associated literature review was also conducted for identifying representative items of other associated potential factors. The entire group of items was reviewed by two measurement and content experts. The scoring system that was applied considered usefulness and non-repetitive content, clarity and appropriateness for parents. Cognitive pre-test interviews were used to test for comprehension and comfort with the response format and instructions. Additionally, for the factors “healthcare professionals’ behaviours” and adherence behaviours, we examined the construct validity and internal consistency reliability using exploratory factor analyses and Cronbach’s, respectively. We also examined internal consistency multi-item scales used for measuring other factors (e.g., knowledge and ability to carry out the home program). Finally, for those factors measured by scales with single items, the reliability was indirectly studied by analyzing the number of non-specific answers or answers left unanswered when not well understood by the patients, resulting in a problem with reliability.

Parents who accepted to participate in this study received the self-administered questionnaire from their attending therapist, who explained how to use the questionnaire and the types of exercises from the checklist, prior to completing the same. The parent more frequently responsible for HEP completed the questionnaire at home and sent it by post to the research team at the University of Murcia. A stamped addressed envelope was provided to encourage response rates, followed by a verbal reminder from the attending therapist one week later. Concurrently, the therapist also collected demographic information regarding the selected parent.

### 2.3. Characteristics of Home Program

The early intervention centres in Murcia commonly provide families with am HEP, so that parents can perform some exercises with their children at home or in everyday situations. Due to the children’s age and condition, HEPs were usually implemented by parents or caregivers. Both the contents of the HEP and the specific dosage of each exercise were previously agreed on between each family and the physiotherapist from the early intervention centre, as instructed by the physiotherapist. The HEPs varied depending on the children’s development, age and needs, including several exercises and instructions. In our study, the questionnaire included a checklist (based on yes/no responses) with three types of therapeutic exercises commonly used in treatments with paediatric populations and explanations about its contents to clarify them to the parents. The three types of therapeutic exercises had been identified from an International Classification of Therapeutic Exercises, as follows [12]: flexibility exercises, neuromotor development training exercises (NDT) (including the training of motor development skills such as head control, crawling, rolling and walking, necessary for improving children’s functioning), and body mechanics and postural stabilisation (BMPS) exercises which included positioning the child with or without external postural support in different positions such as lying, sitting and standing). These types of exercises were recommended to ensure an appropriate alignment of the body segments and the development and control of different postures). 

### 2.4. Adherence Behaviours

Adherence was measured for each type of therapeutic exercise, in accordance with the prescribed dosage of the HEP. We used a five-point frequency-based response scale (never, rarely, sometimes, very often and always), adapted from the adherence scale by Sluijs et al. [13]. The frequency scale had to be answered according to the parents’ adherence to the recommended dosage, in each case. For example, if they had been recommended to perform an exercise once a day, or for 10 min per day, they had to indicate how often they had been able to perform the exercise, compared to the prescription. Other studies [5,13] have suggested that many parents often overestimate their adherence, and we therefore decided to use a highly demanding level in the scale, in order to consider the adherence. Adherence was considered as a dichotomous variable (adherent or not adherent). The categories ‘always’ and ‘very often’ were considered adherent. Furthermore, the same cut-off points were based on the available literature [3,13], and were established before the data analysis. A factor analysis confirmed a one-dimensional structure; the factor explained 60.4% of the total variance, the Kaiser-Meyer-Olkin statistic was 0.61, and the Bartlett statistic was 29.53 (*p* < 0.01). The result of the internal consistency reliability measure was acceptable: α = 0.70.

### 2.5. Potential Factors Associated with Adherence

After reviewing the relevant literature [5,11,14,15], we considered three areas of potential factors: (1) individual; (2) social support and resources; and (3) illness/treatment.

#### 2.5.1. Individual Variables

We assessed parents’ demographic and cognitive variables, as well as children’s demographic variables. Parents’ demographic characteristics were: age (years), gender (male/female), education level (without studies/primary/secondary/university), work participation (yes/no), type of family (two parents/one), and number of children in the home. The child’s age and gender were also measured. 

The following cognitive constructs and instruments were used in the case of parents:

- Perceived barriers to integrate exercises into a daily routine. This was measured using a single item from a validated instrument [13] on a five-point frequency-based response scale (never, rarely, sometimes, very often and always). This scale was coded for statistical analysis as a dichotomous variable, indicating either a low or high perception of barriers, with responses of “never” and “rarely” coded as a low perception.
- Knowledge and ability to carry out the home program. This was measured using two items from a validated parent home program compliance questionnaire [16] (“I understand my child’s home program” and “I am skillful in carrying out...”) on a five-point agreement-based response scale (strongly disagree, disagree, undecided, agree and strongly agree). Low knowledge was defined as a response of “disagree” or less in the two items. We made these transformations before the data analysis and based on previous work [3]. The result of the internal consistency reliability measure was acceptable: α = 0.70.
- Self-efficacy. Parents were asked about how confident they felt participating in the HEP. Measurements were based on a single item from a valid self-efficacy scale [17], with a response scale ranging from 0 (minimum) to 10 (maximum). This measure was categorized on two levels (low or high), with responses equal or higher than the median coded as high self-efficacy.

The non-answer and non-specific answer rate were lower than 5% in all these items (range 1.8% to 4.6%), and therefore they were not excluded.

#### 2.5.2. Factors Related to Social Support and Resources

Social support was measured by a single item indicator assessing the instrumental support provided to the parents on a five-point scale (never-always) and grouped into two categories (low or high), with responses of “very often” and “always” coded as high support. The availability of home equipment for exercises (e.g., exercise mat, ball or other equipment) was reported (yes/no) as a physical resource factor. The non-answer and non-specific answer rate were also lower than 5% (range 1.8% to 2.7%).

#### 2.5.3. Factors Related to Illness/Treatment

We considered the kind of health condition (cerebral palsy/congenital disease/other); functioning (able/not able) for several mobility activities such as sitting, crawling and walking without assistance; and time in treatment (more/less than 2 years).

### 2.6. Healthcare Professionals’ Behaviours during Therapeutic Encounters

Seven relevant questions, based on common paediatric recommendations [3,11,18], were asked concerning three areas: giving general information to parents, instructions and follow-up of exercises. All these questions used a five-point frequency-based response scale (never-always). This measure was categorised on two levels (suitable frequency or not), with the responses ‘always’ and ‘very often’ coded as a suitable frequency. A factor analysis confirmed a one-dimensional structure and therefore the items measured the same underlying construct; the factor explained 59.6% of the total variance, the Kaiser-Meyer-Olkin statistic was 0.86, and the Bartlett statistic was 751.5 (*p* < 0.01). Cronbach’s α was high: α = 0.88; and all of the items contributed to the reliability and construct validity of the questionnaire.

One further item about the parents’ overall satisfaction with the process of care was also included. This was measured using a unidimensional scale ranging from 0 (minimum) to 10 (maximum) and categorised on two levels (low or high), with responses equal or higher than the median coded as a high satisfaction.

### 2.7. Data Analysis

Descriptive statistics using proportions and their 95% confidence interval (CI) were calculated for the description of the sample and the adherence rates. Considering a margin of error (degree of accuracy) of 10% on the estimation of adherence, the minimal sample size required was 96. Respondents and non-respondents to the postal questionnaire were compared by baseline information using the chi-squared test.

To study the relations between the variables, we first examined the association of three groups of potential factors—individual, social and illness factors—associated with the adherence to each type of exercise using univariate and multivariate logistic regression analyses. In the univariate analysis, associations were tested for a significant relationship (*p* < 0.05) with adherence. In the multivariate analysis, the factors with a significant univariate contribution (*p* < 0.10) were combined in a total model for each type of exercise. The parent’s age and gender were always included as independent variables due to their social and psychological relevance. These final models were produced by a process of backwards elimination of independent variables. This procedure consists of dropping an independent variable using the likelihood ratio test at a significance level of *p* = 0.05. The goodness-of-fit and regression for the reduced model were assessed using the methods described by Hosmer et al. [19]. Odds ratios (OR) and 95% CI are reported. 

In a second stage, a multivariate logistic regression analysis of professionals’ behaviour during clinical encounters was used to determine its association with adherence to each type of exercise. Each behaviour and significant variables of the final multivariate first-stage models were introduced as independent variables. Odds ratios and 95% CI are reported for each behaviour in its respective model. The minimal sample size required (*n* = 96) was considered enough for a maximum of five factors in the model, using the recommendation of 10 events (adherent subjects) per variable [20] and considering an estimated 50% of adherence.

## 3. Results

### 3.1. Response Rate and Sample Characteristics

We identified 393 eligible parents from 18 early intervention centres. Of these, 56% returned the questionnaire. Most respondents were women (91%), and most were living with a partner (91%). Table 1 shows the characteristics of the sample. Respondents and non-respondents to the questionnaire did not significantly differ in gender (*p* = 0.850), age (*p* = 0.625), education level (*p* = 0.785), marital status (*p* = 0.584), work status (*p* = 0.233) and number of children (*p* = 0.326). 

Most children were boys (64%) aged between 0.5 and 6 years. 47% were two years old or younger. The most common diagnosis was cerebral palsy (24%) and congenital or hereditary diseases (20%). Almost 70% of children had been receiving early intervention care for less than two years. Most children (94%) received a HEP based on more than one exercise included in the checklist, 51% received prescriptions for flexibility exercises, 70% were prescribed NDT, and 72% were prescribed BMPS exercises.

Some parents failed to respond to the adherence questions regarding flexibility, NDT, and BMPS exercises. Thus, the adherence rates were calculated on 110, 140 and 144 parents, for each of these exercise types, respectively.

### 3.2. Adherence Rates and Associated Factors 

The percentage of parents who adhered to the flexibility exercises was 34%, 50% of parents adhered to the NDT exercises, and 54.2% adhered to BMPS. There was a significant difference between the flexibility and BMPS exercises.

The results of the univariate analyses are presented in Table 2. Based on these results, eight factors were entered into the multivariate model of the flexibility exercises; three factors were entered into the model of the NDT exercises; and five into the model of the BMPS exercises. 

The significant factors in the multivariate models are also presented in Table 3. The model of the flexibility exercises identified knowledge and ability as independent factors. Based on this model, high knowledge increases adherence to flexibility exercises. According to the model of the NDT exercises, a low perception of barriers and high self-efficacy increase the odds of exercise adherence. Finally, according to the BMPS model, the odds of adherence increase when parents have a low perception of barriers and high self-efficacy, as well as when children are unable to maintain an upright sitting position.

Several professional behaviours were identified as significant factors of adherence, although they varied across the different types of exercises. An association was found between the adherence to flexibility exercises and ‘giving information about evolution’, ‘justifying usefulness of treatments’, ‘using the child as a model during instruction of exercises’ and ‘high satisfaction’. Adherence to the NDT exercises was associated with only one professional behaviour: ‘justifying usefulness of treatment’. Adherence to the BMPS exercises was also associated with only one behaviour: ‘asking about adherence at home’.

## 4. Discussion

We examined parents’ adherence to three different types of exercises included in HEPs and its relationship with factors relating to the parents, the child, the environment and the professional. The rates of parents’ adherence to the NDT and BMPS exercises were similar, although the adherence rates for flexibility exercises was lower. This variability among the types of exercises is consistent with the variability reported among therapeutic modalities of medical treatments for children with chronic illnesses [8,21].

In previous studies developed in paediatric populations [5,22], in which the adherence to HEPs was measured as a whole and not separately by exercises, we found similar rates to those obtained for the NDT and BMPS exercises in our study, but higher than our adherence rates for the flexibility exercises. The perception of the overall adherence to HEP, indeed, could overestimate the actual adherence for any specific part of the HEP [16]. 

The associated factors of the adherence to the flexibility exercises were different and more numerous than those associated with others. Adherence to the three types was associated to parental cognitive factors. Nevertheless, whereas the perception of barriers to integrate exercises into daily routine and self-efficacy were common to both the NDT and BMPS exercises, parental knowledge and ability was only associated with flexibility exercises. This may be explained by the fact that these exercises require the use of more manual skills from the parents. The relevance of barriers as an obstacle for adherence is consistent with other studies [6,23], which showed that caregivers only performed home programs if these were easy to integrate into daily family functioning. Self-efficacy has also been identified as critical to sustain the effort of parents with regular advice [24,25]. Moreover, it has been related to adherence to different components of HEP in adults with chronic conditions [26]. 

We found an inverse relation between the adherence to the BMPS exercises and the child’s sitting function, whereas previous studies conducted on children with chronic illness identified that adherence may be lower where there are more functional problems [27,28]. This contradictory effect of functional problems may be because the achievement of specific developmental stages (e.g., sitting) may be more relevant for parents of children with developmental disabilities compared to those with chronic illness. 

The strong relevance of professional behaviours on the flexibility exercises compared to other exercises studied, in our opinion, is consistent with our finding that parents’ knowledge and abilities were strongly associated with the behaviours of professionals. Furthermore, parents usually identify the flexibility exercises as a painful [29,30] and complex [7] activity. This can lead to an inadequate performance of the exercise, different from what the therapist recommended [6,11], or to a reduction in their adherence to this kind of exercises, as our study shows. Thus, it is reasonable to think that when therapists focus on improving parents’ knowledge and abilities, by providing information [23], justifying the usefulness of exercises [2,3] and using the child as a model [3,14], this has a positive effect on adherence. It would be interesting for therapists to consider their influence on adherence to these types of exercises when they plan their interventions with families. 

Understandably, as revealed by studies on the overall adherence to HEPs [11], the variables related to the professional’s interaction and communication with parents are key determinants. In our study, when the parents received information regarding the usefulness of exercises, this significantly increased their odds of adherence both for the flexibility and for BMPS exercises. This could be due to the understanding of a cause-effect relationship, thus improving the adherence rates, especially for those exercises which parents found more difficult to perform. Moreover, in such conditions, where the results from treatments are not immediately observed, a good relationship and communication between parents and therapists could have an important role in the performance of treatments [11]. 

In line with the study by Novak [2], the follow-up of treatments and asking about their performance at home appears to be a strong predictor of adherence to the NDT exercises. Thanks to this follow-up, parents can be invited to explain their difficulties and obtain support from the therapists to overcome barriers and be able to integrate these exercises into their daily routine. However, this follow-up can be perceived in some cases as a source of stress, creating feelings of guilt in parents [18]. Therapists must be aware of this situation, in order to be seen as a source of help instead of as a judgmental onlooker. Other factors, such as the parents’ satisfaction with therapists, should be an additional objective, as this was strongly associated with adherence to the flexibility exercises. Previous studies have also found that satisfaction is relevant to the overall adherence to HEP [18,31,32].

To summarise, the results suggest that adherence is a multidimensional construct, which could be different for each component of the HEP. In fact, parents appreciate that there are different levels of difficulty to be learned between the exercise types [7,14,18]. Therefore, therapists should not categorize parents as being adherent or non-adherent, but should find out their performance for each part of the HEP and their difficulties and barriers for their implementation [33]. More specifically, therapists should be especially attentive when prescribing exercises that parents consider difficult, such as flexibility exercises, or when encountering parents who perceive themselves as poorly skilled. It may therefore be advisable for therapists to spend the time required to make sure that the parents get the skills needed to perform the exercises. It would be recommended to use the child as a model when parents are learning the exercises, especially when these are perceived as difficult. Therapists are also encouraged to inform parents about the usefulness of exercises, as it may not be clear in many types of exercises, also with regards to the child’s evolution. They should also conduct regular follow-ups for the HEP and be aware of parental adherence and difficulties.

Our findings should be interpreted in light of our study’s methodological limitations. First, the cross-sectional design did not allow us to make conclusions about causal effects. Longitudinal studies are needed to draw definitive causal effects and confirm the presence or absence of associations. Second, parents’ behaviours of adherence were self-reported. Although self-reported adherence has demonstrated adequate validity [34], reported levels are the respondents’ perception of their adherence, and are not necessarily a true reflection of actual events. It is therefore possible that our results represent an overly optimistic view of the extent of adherence to HEP. This study also relied on self-reported data regarding health professionals’ behaviours during therapeutic encounters. Third, our questionnaire of parents’ adherence was developed by integrating items from a wide variety of parent-oriented instruments in order to facilitate external validity. The selected items were pilot-tested with parents via cognitive pretesting (unpublished). Parents were asked to report on the relevance and understanding of each item, and all retained items were clearly and well understood by parents. We did not perform an in-depth psychometric evaluation because our study did not use scales from these items; we only examined and verified the internal consistency for the two items used as the scale (knowledge and ability to carry out the home program). Nevertheless, it would be desirable to analyse the factor structure and stability over time in order to ensure the internal validity, especially for questions that are not exhaustive and/or contextual enough (for example, the perceived barriers to integrate exercises into the daily routine, and self-efficacy). Fourth, we initially tested each initial predictor individually with the response and then fit a final multivariate model, only using the variables which were significant for the univariate analysis. This strategy is common and well established, particularly in health sciences; however, it may have statistical consequences, as sometimes insignificant variables in the bi-variate analysis can become significant in a complex multivariate analysis. Fifth, we did not use interaction terms in our multivariate model. Adding interaction terms to a regression model can greatly expand the understanding of the relationships among the variables, and it enables the testing of whether the effect of one predictor variable on the response variable is different at different values of the other predictor variable. Nevertheless, for this study we selected this option because we were only interested in identifying factors associated to adherence. Finally, our sample was recruited from children with or at risk of development disorders attending early intervention centres, who may differ from other children who are in other settings such as hospitals or paediatric clinics. Until further research is conducted, our results should be generalised with caution.

## 5. Conclusions

The rate of adherence between types of exercises is similar, with the exception of the flexibility exercises with lower rates of adherence. Parents’ perceptions of barriers to integrating exercises into their daily routine and self-efficacy contribute significantly to adherence for the NDT and BMPS exercises, and parents’ knowledge and ability to do exercises for flexibility. Physicians and therapists can influence the adherence for all exercises by providing information and follow-up on adherence during clinical encounters and, specifically for the flexibility exercises, by using good practice during exercise instruction. 

Our findings reinforce the need to consider components of the HEP in the management and study of adherence to HEP. Further research is required to identify how interventions to promote adherence to each component contribute to improved clinical outcomes.

## Figures and Tables

**Table 1:** Characteristics of the respondents (*n* = 219).

Variables	N	%
**Gender**		
Male	20	9.1
Female	199	90.9
**Age (years)**		
20–30	47	21.5
31–40	143	65.3
>40	29	13.2
**Educational level**		
Without studies or with primary studies	125	57.1
Secondary studies or university studies	94	42.9
**Marital status**		
With a partner	199	90.9
Without a partner	20	9.1
**Work status**		
Homemaker	114	52.1
Employed	82	37.4
Unemployed	20	9.1
Student	3	1.4
**Number of children**		
1	86	39.3
2	93	42.5
≥3	40	18.2
**Child’s Gender**		
Male	140	63.9
Female	79	36.1
**Child’s Age**		
6 months–2 years	102	46.6
>2 years	117	53.4
**Health condition**		
Cerebral Palsy	52	23.7
Congenital illness *	39	17.9
Other **	128	58.4
**Time in treatment**		
<2 years	156	71.2
≥2 years	63	28.8

* Includes muscular torticollis, spinal atrophy, congenital arthrogryposis multiplex and chromosome motor disorder. ** Includes developmental delay (unspecified), obstetric brachial plexus palsy and encephalopathy in premature babies.

**Table 2:** Odds Ratio (95% Confidence Interval) of predictive factors of adherence to specific types of exercises featured in the home exercise program.

	Adherence to Flexibility Exercises		Adherence to Neuromotor Development Training Exercises (NDT)		Adherence to Body Mechanics and Postural Stabilization Exercises (BMPS)	
	OR Univariate [95% CI] (*n1*)	OR Multivariate [95% CI] (*n* = 97)	OR Univariate [95% CI] (*n2*)	OR Multivariate [95% CI] (*n* = 137)	OR Univariate [95% CI] (*n3*)	OR Multivariate [95% CI] (*n* = 138)
**Parental and Environmental Characteristics**						
**Sociodemographics**						
Age (*n1* = 100) (*n2* = 148) (*n3* = 144)	0.96 [0.90–1.03]		0.99 [0.94–1.05]		0.98 [0.93–1.04]	
Gender Female (*n1* = 100) (*n2* = 148) (*n3* = 144)	1.93 [0.57–6.51]		0.55 [0.15–1.95]		1.01 [0.32–3.18]	
With secondary or university studies (*n1* = 100) (*n2* = 148) (*n3* = 144)	0.40 [0.17–0.95] *		0.71 [0.36–1.38]		0.84 [0.43–1.62]	
With couple (*n1* = 100) (*n2* = 148) (*n3* = 144)	0.76 [0.22–2.68]		0.73 [0.24–2.22]		0.88 [0.29–2.66]	
Work participation (*n1* = 100) (*n2* = 148) (*n3* = 144)	0.46 [0.20–1.05] †		1.14 [0.56–2.31]		1.00 [0.50–2.00]	
Children number (*n1* = 100) (*n2* = 148) (*n3* = 144)						
1	-		-		-	
2	0.61 [0.25–1.50]		0.82 [0.40–1.69]		0.80 [0.39–1.65]	
3 and more	0.90 [0.28–2.91]		0.47 [0.17–1.28]		0.43 [0.16–1.16]	
**Cognitive**						
Low perception of barriers (*n1* = 98) (*n2* = 148) (*n3* = 142)	2.87 [1.20–6.86] *		2.77 [1.38–5.55] **	2.47 [1.16–5.23] *	3.08 [1.5–6.15] **	2.52 [1.15–5.55] *
High knowledge and ability (*n1* = 100) (*n2* = 148) (*n3* = 144)	5.68 [2.2–14.87] **	3.96 [1.35–11.6] **	1.68 [0.86–3.28]		2.59 [1.3–5.11] **	
High self-efficacy (*n1* = 98) (*n2* = 146) (*n3* = 142)	3.28 [1.29–8.32] **		2.62 [1.31–5.24] **	2.21 [1.05–4.66] *	3.98 [1.9–8.28] **	3.69 [1.6–8.34] **
**Environmental factors**						
Support by partner at home (*n1* = 100) (*n2* = 147) (*n3* = 144)	0.95 [0.42–2.16]		1.68 [0.86–3.27]		0.88 [0.46–1.69]	
Having home equipment (*n1* = 100) (*n2* = 147) (*n3* = 144)	1.93 [0.79–4.73]		1.35 [0.63–2.89]		1.45 [0.69–3.05]	
**Child’s Characteristics**						
**Demographics**						
Gender female (*n1* = 100) (*n2* = 148) (*n3* = 143)	1.01 [0.44–2.33]		1.16 [0.58–2.30]		1.67 [0.83–3.36]	
Age: <2 years old (*n1* = 100) (*n2* = 148) (*n3* = 144)	1.16 [0.51–2.65]		1.51 [0.77–2.96]		1.05 [0.54–2.05]	
**Health Related Factors**						
- Health Condition (*n1* = 87) (*n2* = 130) (*n3* = 129)						
Cerebral Palsy	-		-		-	
Congenital illness	2.25 [0.58–8.74]		0.85 [0.28–2.52]		1.17 [0.41–3.34]	
Other	1.55 [0.58–4.15]		0.82 [0.33–2.08]		1.27 [0.53–3.00]	
- Functioning						
Unable to maintain sitting (*n1* = 99) (*n2* = 147) (*n3* = 143)	1.02 [0.40–2.63]		1.19 [0.55–2.58]		2.12 [1.00–4.50] *	2.65 [1.09–6.43] *
Unable to crawl (*n1* = 98) (*n2* = 145) (*n3* = 141)	1.89 [0.81–4.38]		0.72 [0.37–1.42]		1.06 [0.55–2.06]	
Unable to walk (*n1* = 100) (*n2* = 148) (*n3* = 144)	1.27 [0.56–2.87]		1.27 [0.65–2.48]		1.02 [0.52–2.02]	
- In treatment < 2 years (*n1* = 99) (*n2* = 147) (*n3* = 143)	2.17 [0.87–5.42]†		2.20 [0.94–5.16]†		2.14 [0.9–5.12] †	

†: *p* < 0.10; *: *p* < 0.05; **: *p* < 0.01.

**Table 3:** Adjusted Odds Ratio of professionals’ behaviours predictive of adherence to specific types of exercises featured in the home exercise program.

	Adherence to Flexibility Exercises	Adherence to Neuromotor Development Training Exercises (NDT)	Adherence to Body Mechanics and Postural Stabilization Exercises (BMPS)
	OR Multivariate [95% CI] (n1)	OR Multivariate [95% CI] (n2)	OR Multivariate [95% CI] (n3)
**Professionals’ behaviours**			
**Giving information to parents**			
Giving information about evolution (*n1* = 99) (*n2* = 139) (*n3* = 135)	6.27 [1.26–31.16] *	2.62 [0.59–11.55]	1.14 [0.26–5.01]
Justifying usefulness of exercises (*n1* = 97) (*n2* = 141) (*n3* = 137)	9.49 [2.74–32.9] **	2.29 [0.95–5.53]	3.00 [1.20–7.48] *
**Instructions for exercises**			
Giving written instructions (*n1* = 96) (*n2* = 136) (*n3* = 134)	1.43 [0.48–4.25]	1.82 [0.82–4.08]	1.46 [0.62–3.43]
Using the child as a model (*n1* = 99) (*n2* = 141) (*n3* = 137)	2.72 [1.03–7.15] *	2.06 [0.92–4.59]	0.99 [0.42–2.32]
Giving advice to insert into daily routines (*n1* = 97) (*n2* = 140) (*n3* = 136)	1.38 [0.54–3.56]	1.22 [0.60–2.50]	1.01 [0.47–2.16]
**Follow-up of treatments**			
Checking skills (*n1* = 97) (*n2* = 135) (*n3* = 133)	1.55 [0.58–4.15]	1.27 [0.62–2.60]	1.07 [0.49–2.33]
Asking about adherence at home (*n1* = 99) (*n2* = 139) (*n3* = 137)	1.83 [0.70–4.77]	2.98 [1.38–6.41] **	1.42 [0.65–3.13]
Parents’ high satisfaction with the care received (*n1* = 99) (*n2* = 143) (*n3* = 139)	1.71 [1.11–2.64] *	1.14 [0.88–1.48]	1.18 [0.87–1.60]

*: *p* < 0.05; **: *p* < 0.01

## Appendix A

**Table A1:** Questionnaire of Parents’ Adherence to Home Exercise Programs Item scoring, and scales for Questionnaire of Parents’ Adherence to Home Exercise Programs. †

**REGARDING YOUR EXPERIENCE WITH THE HOME EXERCISE PROGRAM**
1. Does the home program fit your daily routine?
2. I understand my child’s home program
3. I am skilful in carrying out the home program
4. How confident are you about performing the home program?
5. My partner supports me at home
6. I have the equipment required to do the exercises at home
**REGARDING THE THERAPIST’S INVOLVEMENT**
7. The physiotherapist gives me information regarding my child’s progress
8. The physiotherapist justifies the usefulness of the exercises
9. The physiotherapist gives me written instructions explaining the exercises
10. The physiotherapist explains the exercises to me using the child as a model
11. The physiotherapist gives me advice on how to include exercises into daily routines
12. The physiotherapist regularly checks my skill at performing the exercises
13. The physiotherapist usually asks me about my adherence to the exercises at home
14. If you had to mark from 1 to 10 your overall satisfaction with the attention from your physiotherapist, what would your score be?
**ADHERENCE BEHAVIOURS**
15. Currently, which of these kinds of exercises have been recommended to you to do at home with your child? □ Flexibility exercises (muscle lengthening, range of motion, stretching) □ Neuromotor development training (head control, crawling, rolling, walking…) □ Posture control training (positioning the child with or without external postural support in different positions, such as lying, sitting, standing…)
16. Many parents find it difficult to do the exercises at home. In your case, how often do you generally do each of the exercises with your child in accordance with the prescribed dosage? □ Flexibility exercises (muscle lengthening, range of motion, stretching) □ Neuromotor development training (head control, crawling, rolling, walking…) □ Posture control training (positioning the child with or without external postural support in different positions, such as lying, sitting, standing…)

† Item statements are presented in the order in which they appeared in the questionnaire. However, the style of the questionnaire is not reproduced here.

Scoring was based on a 5-point Likert scale for items 1, 5, 7–13, 16 (5 always, 4 very often, 3 sometimes, 2 rarely, 1 never), and items 2, 3 (5 strongly agree, 4 agree, 3 undecided, 2 disagree, 1 strongly disagree). Item 4 was scored with a 0–10 point scale (10 very confident–0 not confident at all). Item 6 (1 yes, 2 no). Item 14 was scored with a 0–10 point scale (10 very satisfied–0 very unsatisfied). Item 15 (recommended/ not recommended).

## References

[1] Tinderholt Myrhaug H., Østensjø S., Larun L., Odgaard-Jensen J., Jahnsen R. (2014). Intensive training of motor function and functional skills among young children with cerebral palsy: A systematic review and meta-analysis. BMC Pediatr..

[2] Novak I., Berry J. (2014). Home program intervention effectiveness evidence. Phys. Occup. Ther. Pediatr..

[3] Medina-Mirapeix F., Lillo-Navarro C., Montilla-Herrador J., Gacto-Sánchez M., Franco-Sierra M.Á., Escolar-Reina P. (2017). Predictors of parents’ adherence to home exercise programs for children with developmental disabilities, regarding both exercise frequency and duration: A survey design. Eur. J. Phys. Rehabil. Med..

[4] Psihogios A.M., Fellmeth H., Schwartz L.A., Barakat L.P. (2019). Family functioning and medical adherence across children and adolescents with chronic health conditions: A meta-analysis. J. Pediatr. Psychol..

[5] Başaran A., Karadavut K.I., Üneri S.O., Balbaloğlu O., Atasoy N. (2014). Adherence to home exercise program among caregivers of children with cerebral palsy. Turk. J. Phys. Med. Rehabil..

[6] Hinojosa J., Anderson J. (1991). Mothers’ perceptions of home treatment programs for their preschool children with cerebral palsy. Am. J. Occup. Ther..

[7] Lillo-Navarro C., Medina-Mirapeix F., Escolar-Reina P., Montilla-Herrador J., Gomez-Arnaldos F., Oliveira-Sousa S.L. (2015). Parents of children with physical disabilities perceive that characteristics of home exercise programs and physiotherapists’ teaching styles influence adherence: A qualitative study. J. Physiother..

[8] Dempster N.R., Wildman B.G., Masterson T.L., Omlor G.J. (2018). Understanding treatment adherence with the health belief model in children with cystic Fibrosis. Health Educ. Behav..

[9] Escolar-Reina P., Medina-Mirapeix F., Gascón-Cánovas J.J., Montilla-Herrador J., Valera-Garrido J.F., Collins S.M. (2009). Self-management of chronic neck and low back pain and relevance of information provided during clinical encounters: An observational study. Arch. Phys. Med. Rehabil..

[10] Johnson R.W., Williams S.A., Gucciardi D.F., Bear N., Gibson N. (2018). Evaluating the effectiveness of home exercise programmes using an online exercise prescription tool in children with cerebral palsy: Protocol for a randomised controlled trial. BMJ Open.

[11] Santer M., Ring N., Yardley L., Geraghty A., Wyke S. (2014). Treatment non-adherence in pediatric long-term medical conditions: Systematic review and synthesis of qualitative studies of caregivers’ views. BMC Pediatr..

[12] World Confederation for Physical Therapy (2011). WCPT Guideline for Physical Therapist Professional Entry Level Education. https://www.wcpt.org/guidelines/entry-level-education.

[13] Sluijs E.M., Kok G.J., van der Zee J. (1993). Correlates of exercise compliance and physical therapy. Phys. Ther..

[14] Maring J., Croarkin E., Morgan S., Plack M. (2013). Perceived effectiveness and barriers to physical therapy services for families and children with Friedreich ataxia. Pediatr. Phys. Ther..

[15] Peplow U.C., Carpenter C. (2013). Perceptions of parents of children with cerebral palsy about the relevance of, and adherence to, exercise programmes: A qualitative study. Phys. Occup. Ther. Pediatr..

[16] Law M., King G. (1993). Parent compliance with therapeutic interventions for children with cerebral palsy. Dev. Med. Child Neurol..

[17] Perkins J.M., Multhaup K.S., Perkins H.W., Barton C. (2008). Self-efficacy and participation in physical and social activity among older adults in Spain and the United States. Gerontologist.

[18] Tipping C.J., Scholes R.L., Cox N.S. (2010). A qualitative study of physiotherapy education for parents of toddlers with cystic fibrosis. J. Cryst. Fibros.

[19] Hosmer D.W., Lemeshow S., Sturdivant R.X. (2013). Applied Logistic Regression.

[20] Peduzzi P., Concato J., Kemper E., Holford T.R., Feinstein A.R. (1996). A simulation study of the number of events per variable in logistic regression analysis. J. Clin. Epidemiol..

[21] Kyokunzire C., Matovu N. (2018). Factors associated with adherence to diabetes care recommendations among children and adolescents with type 1 diabetes: A facility-based study in two urban diabetes clinics in Uganda. Diabetes Metab. Syndr. Obes..

[22] Rone-Adams S.A., Stern D.F., Walker V. (2004). Stress and compliance with a home exercise program among caregivers of children with disabilities. Pediatr. Phys. Ther..

[23] McConnell D., Parakkal M., Savage A., Rempel G. (2015). Parent-mediated intervention: Adherence and adverse effects. Disabil. Rehabil..

[24] Desager K., Vermeulen F., Bodart E. (2018). Adherence to asthma treatment in childhood and adolescence—A narrative literature review. Acta Clin. Belg..

[25] Zarei A.R., Jahanpour F., Alhani F., Razazan N., Ostovar A. (2014). The impact of multimedia education on knowledge and self-efficacy among parents of children with asthma: A randomized clinical trial. J. Caring Sci..

[26] Medina-Mirapeix F., Escolar-Reina P., Gascón-Cánovas J.J., Montilla-Herrador J., Jimeno-Serrano F.J., Collins S.M. (2009). Predictive factors of adherence to frequency and duration components in home exercise programs for neck and low back pain: An observational study. BMC Musculoskelet. Disord..

[27] Fiuza-Luces C., Padilla J.R., Soares-Miranda L., Santana-Sosa E., Quiroga J.V., Santos-Lozano A., Pareja-Galeano H., Sanchis-Gomar F., Lorenzo-González R., Verde Z. (2017). Exercise intervention in pediatric patients with solid tumors: The Physical Activity in Pediatric Cancer Trial. Med. Sci. Sports Exerc..

[28] Sil S., Thomas S., DiCesare C., Strotman D., Ting T.V., Myer G., Kashikar-Zuck S. (2015). Preliminary evidence of altered biomechanics in adolescents with juvenile fibromyalgia. Arthritis Care Res. (Hoboken).

[29] Riquelme I., Cifre I., Montoya P. (2015). Are physiotherapists reliable proxies for the recognition of pain in individuals with cerebral palsy? A cross sectional study. Disabil. Health J..

[30] Hadden K.L., LeFort S., OʼBrien M., Coyte P.C., Guerriere D.N. (2015). A comparison of observers’ and self-report pain ratings for children with cerebral palsy. J. Dev. Behav. Pediatr..

[31] Göksan S.B., Bilgili F., Eren İ., Bursalı A., Koç E. (2015). Factors affecting adherence with foot abduction orthosis following Ponseti method. Acta Orthop. Traumatol. Turc..

[32] Essery R., Geraghty A.W., Kirby S., Yardley L. (2017). Predictors of adherence to home-based physical therapies: A systematic review. Disabil. Rehabil..

[33] Miles C., Arden-Close E., Thomas M., Bruton A., Yardley L., Hankins M., Kirby S.E. (2017). Barriers and facilitators of effective self-management in asthma: Systematic review and thematic synthesis of patient and healthcare professional views. NPJ Prim. Care Respir. Med..

[34] Tolley E.E., Guthrie K.M., Zissette S., Fava J.L., Gill K., Louw C.E., Kotze P., Reddy K., MacQueen K. (2018). Optimizing adherence in HIV prevention product trials: Development and psychometric evaluation of simple tools for screening and adherence counseling. PLoS ONE.

